# GDFGAT: Graph attention network based on feature difference weight assignment for telecom fraud detection

**DOI:** 10.1371/journal.pone.0322004

**Published:** 2025-05-30

**Authors:** An Tong, Bochao Chen, Zhe Wang, Jiawei Gao, Chi Kin Lam

**Affiliations:** Faculty of Applied Sciences, Macao Polytechnic University, Macao, China; Federal University of Petroleum Resources Effurun, NIGERIA

## Abstract

In recent years, the number of telecom frauds has increased significantly, causing substantial losses to people’s daily lives. With technological advancements, telecom fraud methods have also become more sophisticated, making fraudsters harder to detect as they often imitate normal users and exhibit highly similar features. Traditional graph neural network (GNN) methods aggregate the features of neighboring nodes, which makes it difficult to distinguish between fraudsters and normal users when their features are highly similar. To address this issue, we proposed a spatio-temporal graph attention network (GDFGAT) with feature difference-based weight updates. We conducted comprehensive experiments on our method on a real telecom fraud dataset. Our method obtained an accuracy of 93.28%, f1 score of 92.08%, precision rate of 93.51%, recall rate of 90.97%, and AUC value of 94.53%. The results showed that our method (GDFGAT) is better than the classical method, the latest methods and the baseline model in many metrics; each metric improved by nearly 2%. In addition, we also conducted experiments on the imbalanced datasets: Amazon and YelpChi. The results showed that our model GDFGAT performed better than the baseline model in some metrics.

## Introduction

With the rapid development of the telecommunication industry, telecommunication fraud has become more and more severe in recent years, and more and more users have suffered property losses. Telecom fraud refers to the behavior of fraudsters who defraud others of large amounts of property through phone calls, text messages, etc [1]. In telecom network fraud, fraudsters will contact many users in order to obtain the personal privacy information of potential victims for further fraudulent behavior. They will step by step, induce the victim to provide personal information and induce the victim to transfer money to a designated bank account [2]. In other cases, the fraudsters lure the victims to open some links to leak their personal information, then the fraudsters get the privacy and steal the money.

In 2017, a survey reported by telecom service providers revealed that losses from telecom fraud amounted to $29.2 billion, accounting for 1.69% of estimated global revenue [3]. In 2023, telecom fraud caused 328.8 billion in losses in China. Telecom fraud damages people’s lives both financially and mentally, even leading to death. Therefore, the fight against telecom fraud has become an urgent global issue [4]. Although governments have taken a number of measures to combat telecom fraud, such as publishing the latest typical fraud cases on social media and using predictive interception mechanisms and systems like China’s National Anti-Fraud Application, cases still occur. Fraudsters continue to improve their strategies, such as disguising themselves as normal users to commit fraud. This makes it very difficult to detect telecom fraud, especially when they disguise themselves as ordinary users in a large number of call records [4].

In the early research on telecom fraud detection, researchers only regarded it as an abnormal sequence detection task, such as using long short-term memory network (LSTM) [5, 6]. In other studies, zhen [4] used the convolutional neural network (CNN) method to convert telecommunication call record (CDR) data into a matrix for research. The methods proposed in these studies have achieved good results, but fraudsters are becoming increasingly intelligent, and their methods are becoming more and more cunning. They will pretend to be normal users to commit fraud and have highly similar characteristics to normal users. Therefore, more than previous methods are needed to deal with today’s fraudsters and explore the complex relationships between them.

**Fig 1:**
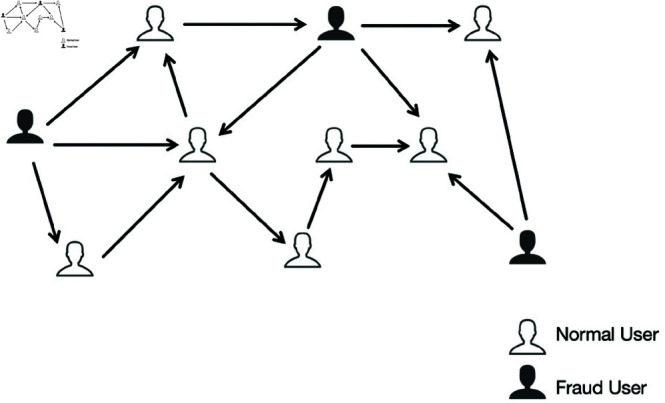
This is an example of a telecom graph network structure. Black users are fraud users, white users are normal users, and the line represents the calling relationship between users.

Current researches propose graph neural network (GNN) methods to address the above challenges. Research based on graph neural networks (GNN) has made significant progress in the field of fraud detection, such as credit card fraud detection [7–9], e-commerce fraud detection [10, 11] and other fields. These successful studies provide new possibilities for telecom fraud. One point that cannot be ignored is that telecom fraud users and normal users are highly interactive and can easily form a graph network structure, as shown in Fig 1. The graph neural network method can aggregate the features of neighbor nodes well and discover potential relationships between nodes. Now graph neural networks have been applied to telecom fraud detection [3, 12–15] etc. The methods proposed in these studies have achieved good results. However, based on our research on these methods and analysis of existing telecom fraud datasets, the application of graph neural networks in the field of telecom fraud detection still has the following difficulties: (1) Telecom fraud users have obvious collaborative relationships, and their characteristics are also irregular. Traditional methods cannot effectively analyze the characteristics. The collaborative relationship between telecom fraudsters usually involves multiple fraudsters working together to defraud a user [12]. In addition, the Sichuan telecom fraud dataset we used has been proven to have collaborative relationships in research such as [12, 16]. (2) In existing research, methods based on graph neural networks update features by aggregating neighbor node features. This makes it impossible to distinguish fraudsters who are highly similar to normal users, further leading to the failure of fraudsters to be discovered.

To solve the above problems, we proposed a novel telecom fraud detection model (GDFGAT) in this paper. For the call detail record (CDR) dataset, we introduce the gated recurrent unit model (GRU) for feature processing, dividing the CDR data into different time windows according to multiple time frequencies. Using GRU for these different time windows can better extract the time series features in each time period and capture the trend or periodic features that may change over time. In addition, we proposed a GAT network that updates based on feature difference weights by calculating the difference weights of the central node and neighbor features. In updating features through GAT message passing, we assign features based on the difference weights, enabling more effective detection of fraudsters and normal users. Considering the node imbalance of fraud detection, we also designed a node degree frequency controller to calculate the frequency weight value of each node through the node degree and use the weight value to alleviate the node imbalance during message passing.

Our contributions are summarized below:

- We used the GRU model to process CDR data from different time dimensions. This model can better extract temporal features within each time period and capture trends or periodic features that may change over time.
- We proposed a new model (GDFGAT) for telecom fraud detection. Based on the GAT model, we innovatively proposed a graph attention network based on feature difference weight update, which can distinguish nodes with highly similar features to better distinguish between fraudsters and normal users. In addition, we also designed a frequency controller based on node degree to alleviate the imbalance of nodes by calculating the frequency weight of each node.
- We conduct extensive experiments on real-world telecom fraud datasets and other public fraud data sets, and the results show that our method outperforms other methods in many indicators.

## Related work

The research of this paper is based on graph neural network telecom fraud detection. In this section, we will introduce the work related to this research.

### Graph neural network

In recent years, GNN has been widely used in many fields, such as natural language processing [17, 18], chemical biology [19–21], recommendation systems [22, 23], fraud detection, causal inference [24, 25] etc. GNN can process graph-structured data and data with complex relationships. It can effectively mine features in the data, thereby better capturing the complex relationships in the graph structure. GNN updates features through the message passing process, where each node receives information from its neighbor nodes and updates its features through an aggregation function. Different graph neural networks also employ different aggregation methods [26].

### Telecom fraud detection

Most of the early work on telecommunications fraud detection was based on traditional machine learning methods. For example, Dominik Olszewski [27] designed a threshold type classification algorithm based on analyzing user features to detect fraudulent users. Amuji [28] proposed a method to group features (number of calls and call duration) to calculate probabilities, then used classifiers and probability models to detect fraud users. Lin [29] proposed a new model (COSIN), a Markov model combined with probability distribution to detect fraud users. Subudhi [30] improved the mean clustering algorithm for telecom fraud detection. Wang [31] extracted call detail record (CDR) data and then used the support vector machine (SVM) algorithm to predict fraud users. Ji [32] used support vector machines with linear, polynomial, and radial basis function kernels to predict fraudulent calls and identify telecom fraud users after modeling the time dimension. Li [33] introduced a state machine into the support vector machine to classify fraudulent users and normal users. However, as the data size grows, the performance of SVM will decline. Other machine learning methods such as decision tree [34], naive Bayes model [35] and ensemble learning [36] have also been applied in the field of telecommunications fraud detection and have achieved good results. However, as fraudsters’ methods become increasingly subtle, traditional machine learning methods cannot effectively detect fraudsters.

### Graph-based telecom fraud detection

In recent years, graph neural networks have been widely used in the field of telecom fraud detection. GNN can learn data with complex relationships and fully mine the relationships between the data. Ji [37] proposed a multi-range gated graph neural network (MRG-GNN) model, which converted some social relationships of users into latent features, then used graph neural networks to learn the latent features of users to detect telecommunications fraudsters and finally achieved good results. Chu [38] used a GraphSage model combined with attention mechanisms to detect fraudsters. Wu [12] proposed a telecommunications fraud detector based on latent collaborative graph learning, which used long short-term memory (LSTM) to encode the original features of user nodes in sequential call behavior learning, constructed a latent collaborative graph by recreating the connections between nodes that share the same call recipient.

## Problem definition

**Definition 1 [Call Detail Record]**. A Call Detail Record (CDR) is a log file generated by a telecommunications company that records information about each call behavior or session. CDR data typically contains information such as call type, call duration, calling and called phone numbers, etc. Each CDR record can be considered a user’s call record sequence. We define the call record sequence of user *u* as $S_{u}=(s_{1},s_{2},s_{3},s_{4},...,s_{L})$.

**Definition 2 [Graph]**. We usually define a simple graph using G=(V,E). Where V represents the nodes in the graph, $V=\{v_{1},v_{2},v_{3},...,v_{N}\}$ can be used to represent all the nodes in the graph. *E* represents the edges in the graph, $e_{ij}=(v_{i},v_{j})\in E$ represents an edge from node $v_{i}\text{to}v_{j}$. This paper we use G=(V,E,X,Y) to define a graph structure for telecommunications fraud detection. Where V represents each node, corresponding to each user, $e_{ij}=(v_{i},v_{j})\in E$ specifically represents that there is a call relationship between user i and user j. *X* represents the feature vector set of the node, defined as $X=\{x_{1},x_{2},x3,...,x_{n}\}$, $x_{i}\in X$ represents the feature vector of node $v_{i}$. *Y* represents the set of node labels, $Y=\{y_{1},y_{2},y_{3},...,y_{N}\}$, where *y*_*i*_ represents the label of node $v_{i}$. In this paper, the value of *y*_*i*_ is defined as $y_{i}\in \{0,1\}$, where *y*_*i*_ = 1 means the node is a fraudulent node, and *y*_*i*_ = 0 means the node is a non-fraudulent node.

**Definition 3 [Telecom fraud detection based on graph neural network]**. The fraud detection problem can be viewed as a classification problem. Using graph methods for fraud detection tasks can convert this task into a graph node classification problem to identify fraud and non-fraud nodes. Graph nodes represent entities that need to be classified. Each node contains corresponding features, and the edges connecting nodes represent the relationship between nodes. Telecom fraud detection can also be viewed as a classification problem to identify whether a node is a fraud node or a non-fraud node. GNN is a neural network that learns graph-structured data and has expressive solid power. GNN aggregates the feature information of neighboring nodes during training and can better capture feature information. Telecom fraud detection based on GNN uses the GNN model to train labeled nodes so that the model can classify unlabeled nodes.

## The proposed method

In this section, we will introduce our model and some key designs.

### Overview

**Fig 2:**
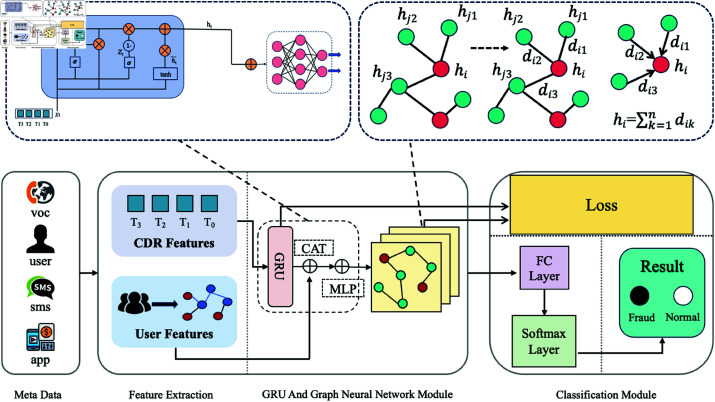
An illustration is provided here to show the details of the proposed GDFGAT model. The model is divided into four modules. (1) Metadata module. (2) Feature extraction module, which extracts features from two data set dimensions. (3) The main model module consists of the GRU model and the GAT model. (4) Classification module, which classifies each node.

Our model GDFGAT consists of four different modules, the structure is shown in Fig 2. The first module is the input metadata information part. The dataset we use comes from the Sichuan Telecom Fraud Competition dataset, which consists of four parts. The Sichuan dataset will be introduced in detail in the next section. The second module is the feature extraction module, which mainly processes the data, extracts features, and constructs graph structure relationships. The third module is the core part of our model, a GRU model and a graph neural network based on feature difference weights. The last module is a classification module that classifies the nodes.

### GRU-based call detail record feature processing

We used the GRU model to process the call detail records (CDR). It is common to use recurrent neural networks (RNN) for time series data, such as call detail records. Considering the memory requirements of recurrent neural networks, we use the GRU model with fewer calculation parameters to improve our performance. We process the CDR data through four different time windows and then input them into the GRU model. This aims to extract as many features of fraudsters as possible in the call time dimension. The calculations for the entire process are summarized as follows:

$$t=(f_{u_{1}},f_{u_{2}},f_{u_{3}},...,f_{u_{n}})\;ts_{i}=GRU(t_{0},t_{1},t_{2},t_{3})(i=0,1,2,3)$$ (1)

Where $f_{u_{1}},f_{u_{2}},f_{u_{3}},...,f_{u_{n}}$ are the call time series features of each user extracted from the call detail records through feature engineering. $t_{0},t_{1},t_{2},t_{3}$ represent four different time windows. We use the features corresponding to each time window as the input of the GRU model and finally obtain the corresponding time series feature information *ts*.

### Feature fusion

In this section, we will briefly introduce the feature fusion method. After obtaining the time series feature *ts*, we concatenate the time series feature and the user behavior feature information *c*, and finally pass an MLP to obtain the final feature *h*. We can express it as follows:

$$c=(c_{u_{1}},c_{u_{2}},c_{u_{3}},...,c_{u_{n}})\;h=MLP(concat(ts_{i},c))(i=0,1,2,3)$$ (2)

Where *h* is the feature after the fusion. *ts*_*i*_ (i=0,1,2,3) is the information of four-time series features, *c* is the information of user behavior features, we concatenate the features of these two dimensions, and finally obtain the total features of each node after fusion through MLP.

### Feature difference weights for graph attention module

In the traditional graph attention network, the feature of the central node is updated by aggregating the features of neighboring nodes. We express the original GAT model feature update as follows:

$$h_{i}^{\prime }=\sigma \left(\sum_{j\in {𝒩}_{i}}a_{ij}Wh_{j}\right)$$ (3)

Where *a*_*ij*_ is the attention coefficient between node i and node j, $h_{j}\in R^{f}$ is the feature of node j, f represents the node feature dimension, and $W\in R^{f\times f^{\prime }}$ represents a learnable weight matrix. *mathcalN*_*i*_ is a collection of neighbors of node i, and σ is a nonlinear activation function. The GAT model can finally obtain the updated feature $h_{i}^{\prime }$ through this calculation formula.

**Our Approach**. Existing GNN models are not explicitly designed for fraud detection, and they cannot effectively identify abnormal states of nodes. Today’s telecom fraudsters are constantly improving their methods and are very similar to normal users in many aspects. Suppose the traditional GNN method is used to aggregate the features between them. In that case, the features of fraud users and the features of normal users will be mixed, and the fraud nodes and normal nodes cannot be effectively identified, resulting in detection failure [26]. Even if the characteristics of fraud users and normal users are highly similar, they cannot be exactly the same, and there must be differences between them. We can compare it to two twins. Even though they look very similar and have similar body shapes, characteristics, and behaviors, there must be differences in specific characteristics. For example, they have different fingerprints. We can identify them by examining the differences between them. Based on this idea, we can calculate the difference weight value of the node and assign features to each node according to the feature difference weight value coefficient in the process of GAT updating features so that the model can more effectively identify fraudulent nodes. The whole calculation process of our method is as follows:

$$hd_{i}^{\left(l\right)}=h_{i}^{\left(l\right)}-h_{j}^{\left(l\right)}\;zd_{i}^{\left(l\right)}=W^{\left(l\right)}hd_{i}^{\left(l\right)}\;e_{ij}^{\left(l\right)}=LeakyReLU({\vec{a}}^{(l{)}^{T}}(W^{\left(l\right)}h_{i}\|zd_{i}^{\left(l\right)}))\;\alpha_{ij}^{\left(l\right)}={softmax}_{j}(e_{ij})=\frac{\exp(e_{ij})}{\sum_{k\in {𝒩}_{i}}\exp(e_{ik})}\;$$ (4)

$$h_{i}^{\left(l+1\right)}=\sigma \left(\sum_{j\in {𝒩}_{i}}\alpha_{ij}^{\left(l\right)}zd_{i}^{\left(l\right)}\right)$$ (5)

Where $hd_{i}^{\left(l\right)}\in R^{f}$ represents the feature difference between node i and node j at layer *l*_*th*_, and $zd_{i}^{\left(l\right)}$ represents the feature difference after linear transformation. $e_{ij}^{\left(l\right)}$ represents the original attention scores of nodes i and j in layer *l*_*th*_, $a\in R^{2F^{\prime }}$ represents a learnable weight vector. First, we concatenate the feature of node i and the feature difference and then transform the dimension by embedding a learnable weight matrix. Finally, use the LeakyRelu activation function to get the original attention scores of node i and node j. $a_{ij}^{\left(l\right)}$ represents the final difference attention weight of node i and node j in layer *l*_*t*_*h*, and $h_{i}^{\left(l+1\right)}$ represents the updated feature of node i.

**Fig 3:**
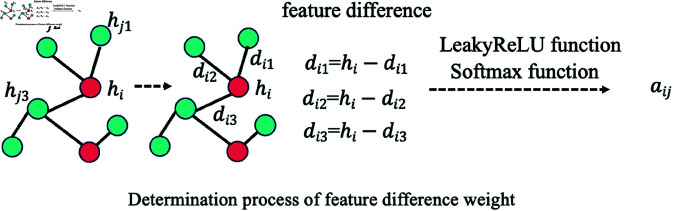
Determination process of feature difference weight.

**Determination process of feature difference weight**. The features *h* of the nodes of each graph network are obtained through the previous process. Refer to Sect 4 Model Structure. Our process of calculating feature difference weights is shown in Eq 4. In the first step, we calculate the feature difference of each node. In Eq 4, the feature difference of each node can be obtained through the first calculation formula. In the second step, a linear transformation is performed. *W* is a trainable matrix, corresponding to the second calculation formula in Eq 4. In the third step, we use the LeakyReLU activation function to get the original attention scores of node i and node j, corresponding to the third formula in Eq 4. Finally, the softmax activation function is used for mapping to get the difference weight between nodes, corresponding to the last calculation formula in Eq 4. The process is shown in the Fig 3 below.

### Frequency controller based on node degree

Fraud detection tasks often face data imbalance. Data imbalance frequently significantly affects the model’s performance, causing minority classes (fraud samples) to be ignored by the model, resulting in detection failure. Graph neural networks can capture complex relationships between nodes. Still, when faced with imbalanced data, the model tends to learn majority class features first, causing it to ignore minority class samples, thus affecting overall performance. The GCN [39] model allocates the feature weights of neighbor nodes through the node degree matrix. Based on this idea, we proposed a node frequency controller method to alleviate the data imbalance. Briefly summarized as follows:

$$norm\_deg(v)=\frac{1}{\sqrt{deg(v)}}$$ (6)

$$g_{ij}=tanh(W\alpha_{ij})$$ (7)

$$K_{ij}=g_{\left(i,j\right)}\cdot (\frac{1}{\sqrt{norm_{d}eg(i)}}+\frac{1}{\sqrt{norm_{d}eg(j)}})$$ (8)

Where Eq 6 is to scale the degree of node v. Here, we refer to the scaling method of the degree matrix in GCN. In Eq 7, W is a matrix for linear changes, *a*_*ij*_ is the difference in attention weight between node i and node j. We adjust the dimension of the attention difference weight and then use the activation function for mapping. Eq 8 sums and normalizes the degrees of node i and node j, then scales again based on the difference in attention weight. The final K value is our balance control coefficient. The final formula of the node update feature is as follows:

$$h_{i}^{\left(l+1\right)}=\sigma \left(\sum_{j\in {𝒩}_{i}}K_{ij}\alpha_{ij}^{\left(l\right)}zd_{i}^{\left(l\right)}\right)$$ (9)

**What is the purpose and principle of what we do?** It can alleviate the problem of data imbalance, thereby reducing the risk of features being biased towards the majority class. First of all, we inverted the degree of each node so that the node with a lot of degrees assigned to each edge of the degree weight is small, and finally, this node can pass very few features of other nodes. Assuming that the degree of the central node is large and most neighboring nodes are normal. This approach can make the weight of each edge very small so that when aggregating features, only a small number of features need to be aggregated, avoiding aggregating all features of normal nodes and finally causing the features to favor most normal nodes. Secondly, we normalize the degrees of the central and neighboring nodes and add them together. This approach balances the influence of the nodes at both ends of the edge to a certain extent. In the message-passing process of the graph neural network, this method can better capture the relationship between the two nodes connected by the edge. When considering the information propagation weight between nodes, this method not only focuses on the degree of a single node but adjusts the propagation weight based on the degree of the two nodes as a whole. This can avoid the situation where only the starting or ending node is biased, making information propagation more reasonable. Finally, the final balance control factor is obtained by allocating according to the attention difference weight coefficient. In the process of feature update, allocating features according to the balance control factor can not only alleviate the impact of imbalance of the data sets to a certain extent, but also ensure that the features are the most effective features when they are transmitted.

**Algorithm 1. Training algorithm.**



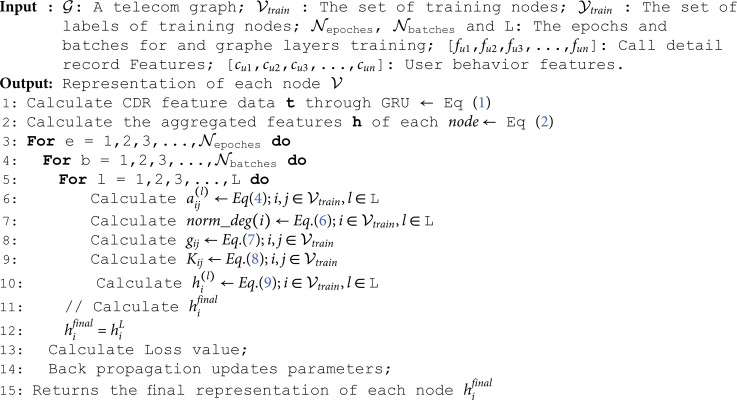



## Experiments

### Dataset

In this paper, we use a real-world telecom fraud dataset provided by the Sichuan Digital Innovation Competition 2020, organized by the Sichuan Big Data Center of China. Due to the large amount of personal privacy involved in telecom fraud data sets, there are few open telecom fraud data sets. Therefore, this section will give a detailed introduction to our dataset. This dataset surveys 6106 users in 23 cities in Sichuan Province, covering a period of 8 months from August 2019 to March 2020. Our analysis of this dataset is summarized in Table 1, which contains 1962 fraudulent users and a total of 5015430 call records. The entire dataset is divided into four parts: user basic information table USER, call behavior table VOC, text message table SMS and APP table. The User table describes basic information about each user, such as phone number, phone bill record, number of phone cards, user label (whether it is a fraudulent user), etc. The VOC table records user call details, including calling number, called number, call type, call date, and call duration features. The SMS table contains user SMS information, SMS type, and the SMS’s date. The APP table records the name of the application and the amount of flow the user uses. The CDR dataset’s feature information is shown in detail in Table 2.

**Table 1:** Sichuan telecom fraud dataset static analysis.

Dataset	Callers	Callees	UserType	Connections
SiChuan	6106	1259878	Normal 4144	
			Fraud 1962	5015430


**Table 2:** CDR dataset features information.

CDR dataset	
feature_name	description
outdegree	Number of calls (calling).Represents the out-degree in the graph nodes
indegree	Number of calls received (called).Represents in-degree in graph nodes
neighbor_degree	Neighbor node degree
recall_rate	Callback rate
coefficient	Clustering coefficient
repeat_rate	Contact repeat rate
mean_dur	Average call duration
var_dur	Call duration variance
call_times_hour*24	Number of calls per hour. The total is divided into 24 hours

**CDR dataset features**. The CDR dataset features used in the project are obtained by feature extraction from the VOC table, as shown in Table 2. The feature dimension size is 32. It should be noted that the feature of the number of calls per hour has a dimension size of 24, and we count it according to the time unit of each hour. Therefore, in our model, the feature size of the GRU module input is also 32, which is consistent with the CDR dataset features.

**Dataset preprocessing**. We perform a unified preprocessing process on the dataset in the project. First, filter and clean the original data set. In this process, we will directly filter out the data with missing features to ensure that the data set is reliable and will not affect the experimental results. The second step is feature extraction and calculation. This process extracts the original features and further calculates other features through the original features to expand the feature dimension size. It will be described in detail in the feature extraction below. The last step is to normalize the features.

**Feature Extraction**. We perform feature extraction on the above datasets and extract features from two dimensions. Extract user call features from the VOC table of user call detail records and extract user behavior features from the remaining three tables. In the VOC table, we extract features such as average call duration, variance, number of calls per hour, user out-degree (total number of calls made), in-degree (total number of calls received), callback rate, and repetition rate. In the text message table, we analyze features such as the frequency and types of text messages sent. In the USER table, we extract features such as the number of user phone cards, average consumption, and variance of consumption. In the APP table, we calculated the total number of apps users use, the total flow, and the average flow features. The feature extraction for each table is summarized in Table 3.

**Table 3:** Features of the Sichuan dataset after extraction.

subtables	Features used
VOC	phone_no_m, opposite_no_m, calltype_id, outdegree*, indegree*, neighbor_degree*, coefficient*, recall_rate*, repeat_rate*, mean_dur*, var_dur*,
	call_times_hour*24, every_one_calltimes*, calls_proportion*, every_different_calltimes*, every_different_ratio*, start_datetime
SMS	phone_no_m, opposite_no_m, sms_calltype, sms_calltype_rate*, sms_count*
USER	phone_no_m, idcard_cnt, arup_201908-arup_202003, arpu_mean*, arpu_var*, arpu_sum*
APP	phone_no_m, flow, flow_mean*, flow_sum*, flow_var*

Features marked with ^*^ are features that we further calculate from the original features.

**Fig 4:**
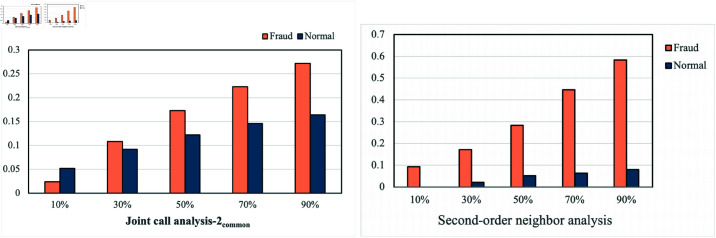
The x-axis on the left side of Fig 4 is the proportion of sample sampling, and the y-axis is the proportion of joint calls made by fraudulent users and non-fraudulent users. The x-axis on the right side of Fig 4 is the proportion of sample sampling, and the y-axis is the proportion of fraudulent users and non-fraudulent users using second-order neighbors or fraudulent users.

**Fig 5:**
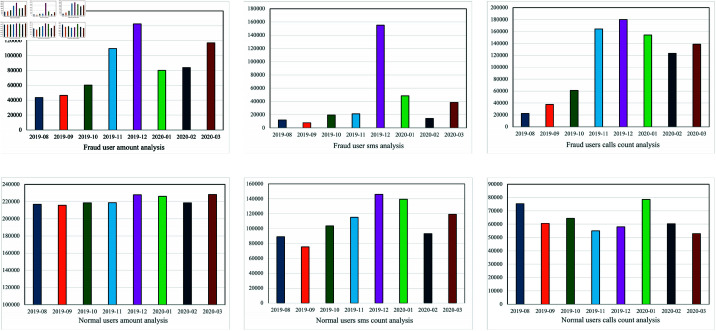
The x-axis represents time, from August 2019 to March 2020, and the y-axis represents the total size of feature statistics of fraudsters or non-fraudsters each month.

**Dataset feature analysis**. The most noticeable feature of this telecom fraud dataset is that the fraudsters have a collaborative relationship. In telecom fraud activities, we define a collaborative relationship as two or more fraudsters working together to commit fraud. In other words, if two or more fraudsters have called the same defrauded person, they may have a synergistic relationship. We sample and analyze the fraudsters in the data. As the proportion of fraudsters continued to increase, the proportion of them calling the same normal user also continued to increase. When the sampling ratio reached 90%, about 30% of the fraudsters called the same user, as shown in Fig 4 left. In addition, we analyze the second-order neighbors of normal users and fraud users, respectively. The results show that 58.3% of the second-order neighbors of fraud users are frauds. However, among the second-order neighbors of normal users, only 8% of users are fraudsters, as shown in Fig 4 right. We can assume that two fraudsters jointly defrauded one user(second-order neighbor), so the collaborative relationship between the fraudsters is very obvious. Secondly, we analyze some features of fraudsters, as shown in Fig 5, and we find that many of the features of fraudsters are collaborative. The features of fraudsters have the characteristic of indirect concentration, which causes their features to show an irregular trend, the trend of feature concentration at a certain time. For example, fraudsters have the highest call charges in December but the lowest in August. Fraudsters are cooperative in some features, leading to the concentration of fraudsters’ features. That is to say, the similarity of fraudsters’ features will increase significantly at a specific point in time. If there is no behaviour synergy, fraudsters’ characteristics should be more random and scattered, with features not concentrated at a particular point in time. This concentration phenomenon indicates that many fraudsters may be engaged in some joint fraudulent operation at these particular points in time, resulting in a concentrated representation of the features.

### Experimental setup

In the experimental section, we conduct extensive experiments on a real-world telecommunications fraud dataset.

**Baseline Methods**. To verify the effectiveness of our proposed method, we compared the GDFGAT model with three types of model methods. The first category is traditional machine learning algorithms. We selected three classic machine learning algorithms: Support Vector Machine(SVM), Logistic Regression(LR) and Random Forest(RF). For the second category, we selected three basic models of graph neural networks, namely the GCN model, the GAT model, and the GraphSAGE model. For the third category, we selected the graph neural network model that has been used in the field of telecommunications fraud detection and fraud detection in recent years.

- **SVM**: Support Vector Machine algorithm is a machine learning algorithm commonly used for classification tasks. It classifies samples by finding a hyperplane.
- **LR**: Logistic Regression algorithm is a machine learning algorithm used for classification tasks. It predicts the probability of an event by establishing a logical function and determines the classification result based on the probability value.
- **RF**: Random Forest algorithm is a machine learning algorithm that can be used for classification tasks, classifying samples through decision trees.
- **GCN** [39]: The graph convolutional network in GNN updates the features of a node by aggregating the feature information of its neighbors.
- **GAT** [40]: Graph Attention Network, which calculates the attention coefficient and performs weighted summation on the features of neighboring nodes to obtain new features of the node.
- **GraphSAGE** [41]: Updates the feature representation of the target node by sampling the neighbors of the node and aggregating the feature information of the neighboring nodes.
- **CARE-GNN** [42]: A graph neural network model for finding informative neighboring node aggregation features based on label-aware similarity metrics.
- **GEM** [43]: A GNN model that uses the attention mechanism to learn the importance of different types of nodes and uses the summation and aggregation features for each node.
- **FRAUDER** [44]: A GNN model with fraud-aware graph convolutional modules to learn discriminative embeddings of normal users and fraudsters.
- **RCGN** [45]: A telecommunication fraud detection method that uses adaptive cost-sensitive learning (AdaCost) to prioritize fraudulent nodes and then uses a deep deterministic policy gradient algorithm to dynamically optimize the weight coefficients.
- **BWGNN** [46]: Proposed a GNN fraud detection model with spectrally and spatially localized bandpass filters to address imbalanced dataset problem.
- **F2GNN** [47]: Proposed a GNN model with adaptive filter with feature segmentation to solve the problem of data imbalance in the field of fraud detection.
- **CDR2IMG** [4]: A computer vision method is proposed to detect telecommunication fraudsters. Convert the CDR data into an image feature matrix, and use the convolutional neural network model to extract the feature matrix.
- **FDAGNN** [26]: A graph neural network method for telecommunication fraud detection with aggregated feature differences.
- **LSG-FD** [12]: A graph neural network telecommunication fraud detector based on latent collaborative graph (LSG) learning.

**Experimental Metric**. We can usually view fraud detection tasks as classification tasks, where we classify fraudsters and non-fraudsters. Therefore, we selected five Accuracy, Precision, Recall, F1 score and AUC metrics to measure our results. Accuracy measures the proportion of correctly predicted samples to the total number of samples. In classification tasks, accuracy is a significant evaluation indicator. Precision represents the fraction of predicted fraudsters that are ground truth. The recall rate represents the proportion of real fraudsters detected. F1 is an indicator that comprehensively considers precision and recall, indicating the trade-off between precision and recall. AUC is used to evaluate the performance and reliability of the classification model. Therefore, this paper mainly evaluates our model from these five metrics.

**Implementation**. During the training process, we selected the Adam optimizer, set the feature embedding dimension size to 32, and set the learning rate to 0.0005. We use T4 GPU on Google Colab to train the Sichuan data set. All experiments use torch-geometric 2.5.3, Pytorch 2.3.0, and Python 3.9. All baselines were implemented using open source code, and our model code can be found on GitHub.

### Performance comparison

As shown in Table 4, our model outperforms other baseline models in all indicators. Among them, the LSG-FD model is the latest and best model for telecommunications fraud detection, and our model also outperforms it in all metrics. We further analyze the experimental results: Firstly, the results obtained by the GNN method are generally better than the traditional machine learning classification algorithms, which also shows that GNN is very suitable for processing data sets with complex relationships. Secondly, our model Accuracy can reach 93.28%, which is about 2% higher than LSG-FD. Compared with this model, our model fully considers the feature change trend of the fraudster and adopts the GRU model to extract features from different time dimensions. This implicitly shows that the proposed method effectively handles the feature concentration caused by the synergistic relationship between fraudsters. FDAGNN is a GNN model that aggregates feature differences. Compared with it, our method is also better than this method in key metrics. Our proposed way of allocating features according to feature difference weights is feasible because GNN has a high sensitivity to scaling factors during the feature update process. There are 1962 fraudulent users in the Sichuan telecommunications fraud dataset, the imbalance rate is 32.13%. The dataset is relatively balanced. To verify the performance of our model on imbalanced datasets, we conducted separate experiments on the Yelp and Amazon datasets.

**Table 4:** Performance comparison of GDFGAT and baseline on SiChuan dataset.

Method	Dataset	SiChuan				
	Metrics	Accuracy	F1	Precision	Recall	AUC
Machine Learning	SVM	0.8627	0.7764	0.7990	0.7710	0.7597
	LR	0.8849	0.8008	0.8576	0.7681	0.8630
	RF	0.8831	0.7787	0.8997	0.7322	0.8666
Classical GNN	GCN	0.8947	0.8921	0.8923	0.8555	0.9289
	GAT	0.8982	0.8955	0.8986	0.8580	0.9288
	GraphSage	0.8465	0.8400	0.8371	0.7920	0.8529
GNN and others	CARE-GNN	0.8742	0.8744	0.9020	0.8748	0.9269
	GEM	0.8488	0.8471	0.8852	0.8490	0.8904
	FRAUDRE	0.8928	0.8683	0.8354	0.8452	0.8912
	RGCN	0.9094	0.9072	0.9111	0.8730	0.9393
	BWGNN	0.8936	0.8738	0.8786	0.7755	0.9379
	F2GNN	0.8756	0.8598	0.8933	0.7262	0.9031
	CDR2IMG	0.9033	0.8998	0.9085	0.8924	0.9298
	FDAGNN	0.9151	0.8819	0.9182	0.8744	0.9211
	LSG-FD	0.9189	0.9043	0.9206	0.8945	0.9318
Ours	GDFGAT	**0.9328**	**0.9208**	**0.9351**	**0.9097**	**0.9453**

## Model comparison on imbalanced dataset

The Sichuan dataset mentioned above shows that fraud and non-fraud users are relatively balanced. So, in this section, we perform an imbalanced test on the model to verify that our model also performs well on imbalanced data sets. We use data sets from Amazon and Yelp, two publicly available data sets in the field of fraud detection. The Amazon dataset collects user reviews under the category of musical instruments. There are three connections between users, products, reviews, and time. It connects users who have reviewed at least one of the same products (U-P-U) and have at least one review with the same star rating within a week. User (U-S-U) means the top 5% users among all users with similar text comments to each other (U-V-U). The Yelp dataset comes from spam or deceptive reviews of restaurants or hotels. There are also three connections, linking reviews posted by the same user (R-U-R) with the same star rating (R-S-R) and the same product in the same month (R-T-R). The analysis of Amazon and Yelp data sets is shown in Table 5.

**Table 5:** Static analysis of Yelp and Amazon data sets.

Dataset	Nodes	Relations	Edges	IR
Amazon	45954	R-U-R	49315	0.145
		R-T-R	573616	
		R-S-R	3402743	
Yelp	11944	U-P-U	3566479	0.095
		U-S-U	175606	
		U-V-U	1036737	

**Baseline Methods**. To validate our proposed method, we conducted extensive experiments on two imbalanced data sets, Amazon and Yelp. We selected some recently proposed models in fraud detection (including some models from the previous experiment) and models used to solve the problem of imbalanced dataset fraud detection as our baseline.

- **GHRN** [48]: A GNN fraud detection model based on spectral analysis.
- **PC-GNN** [49]: A GNN model based on resampling method to solve the problem of data imbalance.
- **GDN** [50]: A graph decomposition network that infers and updates the distribution of abnormal features through prototype vectors for the field of graph anomaly detection.

**Experimental Metric**. For two imbalanced data sets, Amazon and Yelp, we used F1, AUC, Recall, and G-Mean to measure the performance of our model on imbalanced data sets. G-Mean metric means the geometric mean. When evaluating imbalanced data sets, we usually measure the size of the G-Mean value. G-mean can better compare the performance of different models on imbalanced data sets.

### Performance comparison on imbalanced dataset

Table 6 shows the results of our experiments on imbalanced data sets. Our model also performs well on imbalanced data sets. Compared with the results of the Sichuan dataset, we can find that the models generally perform worse. For example, the AUC and F1 of the CARE-GNN model are much lower, and the impact of data imbalance on the model is evident. Compared with the LSG-FD model on the Amazon dataset, our model has a lower AUC but is better than that model in other indicators. On the Yelp dataset, the G-mean metric is lower than the GDN model, but other metrics are better than other models. Our model also performs well on imbalanced data sets in other metrics compared to other models.

**Table 6:** Performance comparison of GDFGAT and baseline on Amazon and Yelp dataset.

Method	Dataset	Amazon				Yelp			
	Metrics	F1	AUC	Recall	G-Mean	F1	AUC	Recall	G-Mean
Baselines	PC-GNN	0.8480	0.9626	0.9128	0.9098	0.6680	0.8284	0.7508	0.7260
	BWGNN	0.9084	0.9769	0.7924	0.9021	0.7174	0.8458	0.5509	0.7726
	GHRN	0.9194	0.9395	0.8037	0.9058	0.7689	0.8383	0.5174	0.7348
	CARE-GNN	0.8797	0.9459	0.8694	0.7114	0.6246	0.7878	0.7175	0.6919
	FRAUDRE	0.9110	0.9345	0.8894	0.8835	0.6359	0.7548	0.6930	0.7157
	F2GNN	0.9192	0.9783	0.8354	**0.9162**	0.7942	0.9230	0.4352	0.6998
	GDN	0.9085	0.9794	0.8951	0.9066	0.7554	0.8941	0.7912	**0.8087**
	LSG-FD	0.9216	**0.9808**	0.9152	0.8976	0.7737	0.9216	0.8362	0.7220
Ours	GDFGAT	**0.9230**	0.9704	**0.9250**	0.9116	**0.7996**	**0.9314**	**0.8571**	0.7470

### Quantitative description of experimental results

In the above experimental part, we conducted experiments on the model on relatively balanced data sets and unbalanced data sets. On the Sichuan dataset, the accuracy of our model GDFGAT reaches 93.28%, while the SVM, LR, and RF in traditional machine learning algorithms are 86.27%, 88.49%, and 88.31%. Among the classic GNN models, GCN is 89.47%, GAT is 89.82%, and GraphSage is 84.65%. Among models in the same field, the latest LSG-FD model is 91.89%. Our model is 5% higher than the traditional machine learning algorithm, about 4% higher than the traditional GNN method, and about 2% higher than the latest method LSF-FD. In terms of F1 score, GDFGAT is 92.08%, SVM is 77.64%, LR is 80.08%, and RF is 77.87%. Our method improves 12% over the machine learning method. Among traditional GNN methods, the best performing method is GAT with 89.55%, and our method has an improvement of about 3%. Among related research methods, the best performing method is LSG-FD with 90.43%, which is about 1.65% higher than LSG-FD. In terms of Precision, GDFGAT is 93.51%, RF is 89.97%, and LSG-FD is 92.06%. GDFGAT is about 4% higher than RF and about 1.45% higher than LSG-FD. In terms of Recall, GDFGAT is 90.97%, SVM is 77.10%, and LSG-FD is 89.45%. GDFGAT is about 13.87% higher than SVM and about 1.52% higher than LSG-FD. Similarly, in terms of AUC, GDFGAT is 94.53%, RF is 86.66%, and LSG-FD is 93.18%. GDFGAT is about 8% higher than RF and about 1.35% higher than LSG-FD. The above comparison indicators clearly demonstrate the advantages of the GDFGAT model in various metrics. In the imbalance experiment, we used Amazon and Yelp datasets. Considering that imbalance has a great impact on accuracy, we used F1 score, AUC, Recall and G-mean as indicators for testing. For the Amazon dataset, the imbalance rate is 14.5%. Compared with the baseline model, GDFGAT is 92.30% in F1 score, while the best performing method among other methods is LSG-FD, which is 92.16%. In comparison, our method has improved by about 0.2%. In Recall, it is 1% higher than LSG-FD.

For the Yelp dataset, the imbalance rate is 9.5%. In F1 score, GDFGAT is 79.96%, which is about 2% higher than the best LSG-FD among other methods. In AUC indicator, GDFGAT is 93.14%, which is about 1% higher than the best other methods. In Recall indicator, GDFGAT is 85.71%, which is about 2% higher than other methods.

The results show that on a relatively balanced dataset, our model GDFGAT performs best on all metrics. Considering the impact of imbalance problems on the model, even so our model is better than other methods in some metrics, and is also very close to existing advanced methods in some indicators, such as the G-mean metric. The imbalance test also shows that our model GDFGAT has a good performance on imbalanced datasets.

## Ablation experiments

**Fig 6:**
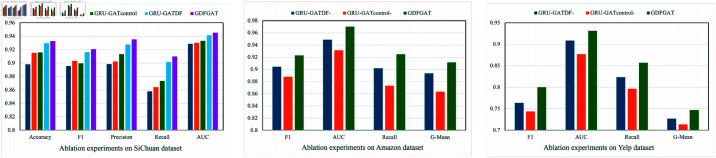
Ablation experiment diagram on SiChuan, Amazon and Yelp data sets. The x-axis represents each metric and the y-axis represents the corresponding value. The SiChuan dataset has five metrics, and the Yelp and Amazon data sets have four metrics.

To illustrate the effectiveness of each module of our GDFGAT model, we conducted multiple sets of ablation experiments. We designed the following variant models for experiments: (1). The original GAT model. (2). Introduce the GRU module to verify its validity (GRU-GAT). (3). Add the feature difference weight algorithm to verify the effectiveness of the algorithm (GRU-GAT_*DF*_). (4). Introduce the frequency controller based on the node degree module and verify the effectiveness of the module (GRU-GAT_*control*_). We also conducted ablation experiments on the feature difference weight algorithm and the degree-based frequency controller module on the Amazon and Yelp datasets. The variant models are as follows: (1). The variant model GRU-GAT_*control*−_ that ignores the degree-based frequency controller module. (2). The variant model GRU-GAT_*DF*−_ that ignores the feature difference weight algorithm. We conducted ablation experiments on the Sichuan, Amazon, and Yelp data sets, and the results are shown in Fig 6.

As seen from the results in Fig 6, our model performs best on Sichuan, Amazon, and Yelp data sets. From this dimension, our model is more effective than other models. On the Sichuan dataset, the results of the variant model GRU-GAT_*DF*_ have good performance and are also very close to our final model. The reason for this phenomenon is that the updated feature algorithm based on the weight assignment of the feature difference we propose has a good effect. The performance of variant GRU-GAT_*control*_ is close to that of variant GRU-GAT, probably because our proposed frequency controller based on node degree does not work well. The reason may be that the Sichuan dataset is relatively balanced. Our assumption is verified on Amazon and Yelp data sets. The results of the variant model GRU-GAT are better than the original GAT model, so the GRU module we introduce effectively processes the call feature behaviors of different windows. The performance of the original GAT model on the Sichuan dataset is lower than that of other variant models. On the Yelp dataset, after deleting the method of frequency controller based on node degree, GRU-GAT_*control*−_ performs poor than other variant models. Considering the imbalance of the Yelp dataset, our proposed method to alleviate data imbalance is effective on imbalanced data sets. Similarly, on the Amazon dataset with a lower imbalance rate, the variant model GRU-GAT_*control*−_ still performs poorly, and its various indicators are lower than other variant models.

### Parameter sensitivity

**Fig 7:**
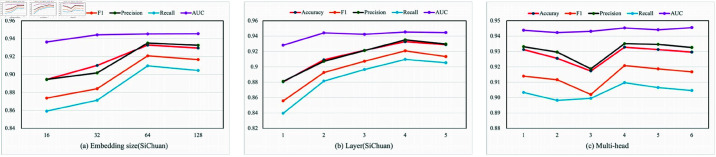
Performance of GDFGAT with different embedding sizes, layers, and multi-head on the Sichuan dataset. In Fig(a), the x-axis represents the embedding size, and the y-axis represents the corresponding value. In Fig(b), the x-axis represents the number of graph neural layers, ranging from 1 to 5, and the y-axis represents the corresponding value. In Fig(c), the x-axis represents the number of heads of the graph neural network, ranging from 1 to 6, and the y-axis represents the corresponding value.

In this section, we conduct experiments on the GDFGAT model’s key parameters to explore the model’s sensitivity to the parameters. We study the sensitivity of embedding size, the number of layers in the graph attention network, and the number of heads in the graph attention network, the result is shown in Fig 7. Among them, the embedding size ranges from 16 to 128, as shown in Fig 7(a). When the range increases from 32 to 64, the indicators are significantly improved. However, when the embedding size exceeds 64, the performance begins to decline. We need to pay extra attention to the fact that the larger the embedding size, the higher the computing requirements, and the operating efficiency will also slow down as the embedding size increases. Considering the efficiency, we set the embedding size to 64. As shown in Fig 7(b), the number of layers of the graph attention network ranges from 1 to 5. When the number of layers of the graph attention network increases from 3 to 4, the performance improves significantly, and when 4 increases to 5, the performance reaches a steady state. We also need to pay extra attention to the fact that the number of layers also affects the efficiency of the operation, so we finally set the number of layers to 4. Fig 7(c) shows the multi-head size of the graph attention network, ranging from 1 to 6. When the multi-head size is 4, the model performs best. When the size exceeds 4, the performance begins to decline, so we set the multi-head size to 4.

## Conclusion and future work

In this paper, we proposed a novel model called GDFGAT for telecom fraud detection. In the GDFGAT model, we introduce the GRU module to process call characteristics in different time windows. In the graph neural network module, we proposed a new graph attention network model DIFFGAT based on the node feature difference weights based on the graph attention network model and update the features according to the feature difference weights of the central node and the neighboring nodes. Finally, we design a degree frequency controller according to the node’s degree information to alleviate the problem of data imbalance. We conducted a large number of experiments on real telecommunications fraud datasets and two public fraud datasets, and the results show that our model GDFGAT has a good performance. GDFGAT has made progress in the direction of telecom fraud detection, but limited by the dataset, our model can only be validated on the Sichuan dataset at present. Therefore, in future work, we will seek more complex telecom fraud data sets for experiments to improve our model. In addition, we also plan to conduct further research on the problem of data imbalance and propose more effective solutions. Finally, we will study applying the model to other fraud detection directions.
